# MicroRNA as Type I Interferon-Regulated Transcripts and Modulators of the Innate Immune Response

**DOI:** 10.3389/fimmu.2015.00334

**Published:** 2015-07-08

**Authors:** Samuel C. Forster, Michelle D. Tate, Paul J. Hertzog

**Affiliations:** ^1^Centre for Innate Immunity and Infectious Diseases, Hudson Institute of Medical Research, Clayton, VIC, Australia; ^2^Department of Molecular and Translational Sciences, Monash University, Clayton, VIC, Australia; ^3^Host-Microbiota Interactions Laboratory, Wellcome Trust Sanger Institute, Hinxton, UK

**Keywords:** microRNA, interferon, post-transcriptional regulation, systems biology, interferon-regulated genes

## Abstract

Type I interferons (IFNs) are an important family of cytokines that regulate innate and adaptive immune responses to pathogens, in cancer and inflammatory diseases. While the regulation and role of protein-coding genes involved in these responses are well characterized, the role of non-coding microRNAs in the IFN responses is less developed. We review the emerging picture of microRNA regulation of the IFN response at the transcriptional and post-transcriptional level. This response forms an important regulatory loop; several microRNAs target transcripts encoding components at many steps of the type I IFN response, both production and action, at the receptor, signaling, transcription factor, and regulated gene level. Not only do IFNs regulate positive signaling molecules but also negative regulators such as SOCS1. In total, 36 microRNA are reported as IFN regulated. Given this apparent multipronged targeting of the IFN response by microRNAs and their well-characterized capacity to “buffer” responses in other situations, the prospects of improved sequencing and microRNA targeting technologies will facilitate the elucidation of the broader regulatory networks of microRNA in this important biological context, and their therapeutic and diagnostic potential.

## Introduction

**Figure 1:**
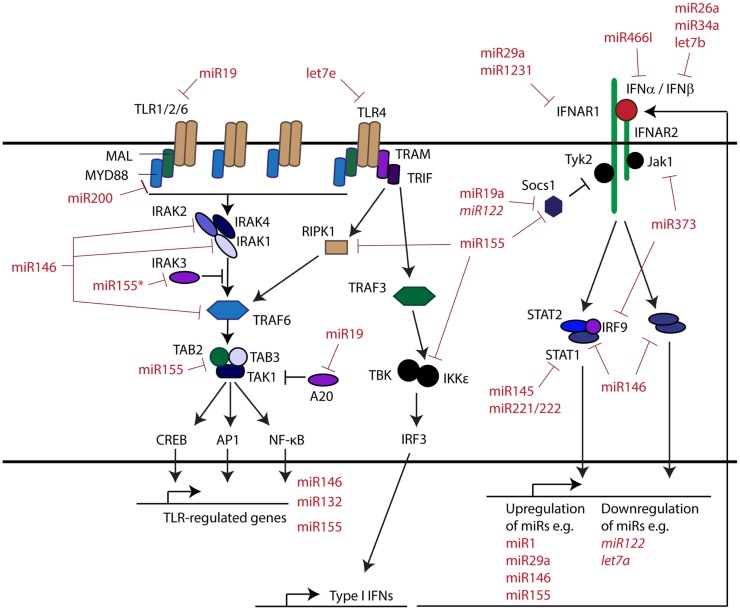
**MicroRNAs and their roles in regulating the innate immune response**.

The innate immune system provides the first line of defense against invading pathogens and plays a vital role in the detection of cellular disturbances. This system is initiated through activation of pattern recognition receptors (PRRs), such as the Toll-like receptors (TLRs) (1), Nod-like receptors (2), and RIG-I like helicases (3), which act as a sophisticated detection network, recognizing danger signals and initiating both intra- and intercellular responses. Importantly, the intercellular responses regulated through these pathways act to recruit and guide the broader immune response. The PRR intracellular signaling pathways are composed of well-characterized components including adaptors (e.g., MyD88, TAB) and enzymes (e.g., IRAKs and IKKs) that activate two main transcription factors, namely NF-κB, which drive pro-inflammatory cytokine gene transcription, and the IRFs, which drive expression of type I interferon (IFN) gene transcription (Figure 1). The type I IFNs are inducible cytokines that play an important role in many aspects of immunity (4) and have been shown to regulate over 2000 coding and non-coding large RNA transcripts, termed IFN-regulated genes (IRGs). This regulation occurs in a highly coordinated manner, the exact nature of which is dependent on subtype, timing, dosage, cell, and pathophysiological context (5).

At the systems level, an understanding of signaling in the innate immune response has been initiated (6, 7). Over the last decade, a role for particular microRNAs and other non-coding RNA in sculpting and modulating many levels of the innate immune response has begun to emerge (8). This includes targeting transcripts encoding components of PRR pathways impacting IFN production, targeting transcripts encoding the IFN cell surface receptors and signal transduction proteins to regulate signaling, and targeting IRGs directly, to shape the overall IFN response. At each of these levels, microRNAs may suppress activation by targeting key signaling components or enhance signaling, by suppressing negative regulators. Interestingly, microRNAs themselves may also be induced or repressed directly through IFN signaling, introducing an additional layer of regulation to this response. This review will highlight the growing body of research in this area.

## MicroRNA Biogenesis and Function

Unlike many families of non-coding RNAs, the processing, accessory proteins and functional requirements for microRNA activity are relatively well understood. Processing from a primary to a mature microRNA occurs through a series of cleavage events dependent on the enzymes Drosha, Dicer, and associated accessory proteins (9). Through this processing, one of the two precursor hairpin RNA strands is incorporated into the RNA-induced silencing complex (RISC) containing the Argonaute (Ago) proteins. These mature microRNA contained within the RISC complex are small, approximately 21–23 base pair transcripts. Through nucleotide homology, they bind the target messenger RNA (mRNA) molecule, usually within the 3′ untranslated region (UTR), to direct RNA silencing. This silencing has been shown to occur through mRNA cleavage, transcript destabilization by shortening of the mRNA polyadenylated tail, or through direct target degradation (10).

Initial work defining microRNA function has focused largely on constitutively expressed and cell-specific microRNA, assigning to these microRNAs important functions in cell differentiation, lineage commitment, and the determination of cell fate (11, 12). High-throughput expression profiling studies of microRNAs in the context of cancer-related diseases, for example, have demonstrated microRNA dysregulation, predominantly reduced expression (13). These observations have led to a model suggesting that microRNAs act primarily in determination of cell fate by guiding differentiation and maintenance of homeostasis. Further experiments using gene profiling have demonstrated a capacity to classify cancers by microRNA expression with many studies investigating the use of microRNAs as circulating biomarkers for prognostic and diagnostic purposes, as recently reviewed in Ref. (14). There is now growing knowledge of the role played by microRNA regulation in other biological contexts, including in protecting against infection and regulating the immune response (8, 15).

## MicroRNA Regulation of Innate Immune Signaling Leading to IFN Production

Activation of a number of PRR signaling pathways may result in the production of a subset of IFNα subtypes, the single IFNβ subtype or both a subset of IFNα subtypes and the IFNβ subtype in combination. While there are no reported stimuli that activate only a single IFNα subtype, nor all IFNαs, IFNβ is expressed exclusively in circumstances including M-CSF stimulated macrophage progenitors and RANKL stimulated osteoclast progenitors (16). All IFN subtypes commonly bind the IFNAR receptor complex and activate similar signal transduction pathways, although IFNβ can also initiate an additional transcriptional response through a unique receptor interaction (17). With increased microRNA expression profiling of innate immune pathways, a growing number of inducible microRNAs are being described that regulate signaling pathways that lead to IFN production. MiR146 and miR155 are two well-described, highly inducible microRNAs, which were initially identified by microarray on human THP-1 cells as responsive to the TLR4 agonist LPS (18). Extensive characterization demonstrates rapid induction of these microRNAs in response to activation of many innate immune pathways, including those initiated by TLR2, TLR4, TLR5, TNFα, and IL-1β (18). Fluorescence reporter and microRNA decoy assays have also demonstrated miR146 targeting of TLR signaling molecules, MAL, TRAF6, IRAK1, and IRAK2, which are involved in initiating NF-κB responses (19, 20).

Studies using peripheral blood mononuclear cells (PBMCs) from patients with systemic lupus erythematosus (SLE), a disease commonly associated with excessive type I IFN signaling, identified significant down-regulation of miR146 in PBMCs from patients with this disease (21). Under-expression negatively correlated with clinical disease activity and with IRG expression, while transfection of miR146 transcriptionally suppressed IFNα and IFNβ expression. In an Epstein–Barr Virus positive lymphoma cell line, miR146 targeted both STAT1 and IRF5 transcripts (21) and resulted in modification of the intensity of the IFN response through suppression of IRGs (22). Given this regulatory relationship, in which miR146 is both induced by NF-κB and acts to negatively regulate NF-κB and IFN signaling, an important negative feedback relationship is suggested. Indeed, mice lacking miR146 exhibit an autoimmune disease phenotype and over-activation of NF-κB signaling pathways in a manner consistent with this mode of action (23, 24).

Strong up-regulation of miR155 in response to LPS activation of TLR4 is well described (18, 25, 26). A recent study demonstrated both NF-κB and ETS2 play a key role in the regulation of miR155 (27). In the context of TLR signaling, western blot and 3′ UTR luciferase assays have demonstrated miR155 targeting of TNF adaptor molecules Fas-associated death domain protein (FADD), the serine-threonine kinase Ripk1 and IkappaB kinase epsilon (IKKɛ) (28), c-Fos (29), the signaling molecule TAB2, and the transcription factor PU.1 (30). Multiple targets within TLR pathways suggest a role for miR155 in providing protection against uncontrolled TLR responses (28). Significant investigation has also been performed contrasting the actions of the alternate miR155 minor strand during TLR7 signaling (31), suggesting coordinated regulation of the miR155* strand provides a mechanism to modulate IFN production in this response. Rapid up-regulation of miR155* suppresses translation of the negative regulator of TLR signaling, IRAK3, thus potentiating signaling and resulting in greater induction of both TNFα and IFN (31, 32). This signal enhancement mediated by miR155* contrasts with the buffering role provided by miR155, suggesting a need for further investigation into the expression and activation profiles of the two miR155 strands.

Other microRNAs have been associated with regulating PRR signaling pathways and thus may play important roles in regulation of the IFN response. TLR2 and TLR4 have been shown to be regulated by miR19 (33) and let7e (34), respectively, while the important adaptor molecule MyD88 is targeted by the miR200 family (35). Since the pathways in which these receptors and adaptor are found are involved in the induction of IFN gene expression, these microRNAs may thus impact on IFN production, but this has not been directly shown. These data together suggest a complex regulatory network interacting to balance the degree and duration of the TLR and thus IFN responses.

Multiple members of the *IFN*α gene family have been shown to be directly targeted by miR466i at the transcript level, reducing IFN production by macrophages and dendritic cells, as well as the induction of an anti-viral response (36). Similarly, miR26a, miR34a, and let7b directly target the *IFN*β** gene transcript. Interestingly, IFNβ signaling was shown to up-regulate the expression of these microRNAs, indicating a potential negative feedback loop to buffer IFNβ production (37). Overall, these studies demonstrate an important, emerging role for microRNA regulation of IFN production, which may be applied directly through targeting IFN transcripts or indirectly through subtle manipulations of the strength, timing, and duration of the upstream PRR signaling pathways.

## MicroRNA Regulation of Type I IFN Signaling

In addition to impacting IFN production through targeting PRRs, there is mounting evidence that microRNAs also directly regulate different aspects of the type I IFN signaling pathway. Type I IFNs bind to the cell surface IFNAR receptor complex, comprised of the subunits IFNAR1 and IFNAR2, resulting in phosphorylation and activation of the associated tyrosine kinases TYK2 and JAK1 (Figure 1). These in turn phosphorylate receptor tyrosine residues leading to the recruitment and activation of transcription factors. These transcription factors include STAT1, STAT3, and STAT5 homo- and heterodimers, the ISGF3 complex, composed of STAT1, STAT2, and IRF9, as well as STAT-independent pathways (16).

At the type I IFN receptor level, multiple microRNAs have been reported to target IFNAR1. MiR29a has been shown experimentally to reduce the expression of IFNAR1 on murine thymic epithelial cells, reducing IFN responses critical in the regulation of thymic cellularity (38). In this study, mice lacking miR29a displayed increased thymic IFNAR1 expression and hyper-sensitivity to polyI:C treatment. A single nucleotide polymorphism (SNP) ablating miR1231 regulation of IFNAR1 has been associated as a causative factor in hepatocellular carcinoma (39). MicroRNAs can also indirectly modulate type I IFN signal transduction by targeting SOCS1, a negative regulator of the JAK-STAT pathway, which binds the receptor complex through interaction with TYK2 (40). MiR19a, miR122, and miR155 have been shown to target SOCS1, resulting in enhancement of type I IFN signaling and subsequent innate and adaptive immune responses (41–45). This miR155 targeting of SOCS1 is consistent with increased phosphorylation of STAT1 and STAT3, resulting in the enhanced induction of anti-viral genes and inhibition of HBV replication (46).

Downstream of the IFN receptor, STAT1, STAT2, and STAT5 are targeted by a number of microRNAs, as reviewed previously (47). In particular, STAT1 is targeted by miR145, miR146, and miR221/222, and STAT2 by miR221/222, reducing type I IFN signaling and IRG expression. A recent study also illustrated that miR373 reduces the expression of both JAK1 and IRF9, leading to reduced type I IFN anti-viral gene induction and increased HCV replication (48). STAT3 regulation by microRNAs, including miR9, miR93, miR20a, and miR17, has also been examined in many disease and developmental contexts, as previously reviewed in Ref. (49). In the context of IFN response, the direct regulation of STAT3 remains to be elucidated.

## IFN Regulation of MicroRNAs

While extensive characterization of the microRNA response to type I IFN examining temporal or subtype variation has not been reported, analysis using microarray based expression profiling has provided some insight into IFN-regulated microRNAs. Characterization of IFNβ-regulated microRNAs in Huh7 cells, with a custom microarray containing 245 microRNAs from humans and mice, identified 30 microRNAs that were induced or suppressed (50). Interestingly, these included eight induced microRNAs (miR1, miR30, miR128, miR196, miR296, miR351, miR431, and miR448) that displayed complementarity in their seed sequences with hepatitis C virus RNA. In addition, miR122, which positively regulates HCV replication, was suppressed by IFNβ. MiR122 acts to enhance viral replication by shielding the HCV genome from the cytosolic RNA exonuclease, Xrn1 mediated degradation, and another, yet undefined, Xrn1 independent mechanism (51). Other studies in human glioma cells stimulated with IFNβ used more advanced microarrays containing 662 probes. These experiments identified induction of miR187 and miR194 and suppression of miR100, let7a, let7b, let7c, and miR21 (52). In total, 36 type I IFN-regulated microRNAs have been reported to date using arrays and quantitative real-time PCR (Table 1). Of these, 18 are regulated by IFNβ, 14 by IFNα, and 4 have been shown to be regulated by both IFNβ and IFNα. While 21 microRNAs are reported to be up-regulated after IFN stimulation, 13 are suppressed. Let7b and miR30 are regulated differentially in a cell type-specific manner (50, 53, 54). Interestingly, this list includes a number of key microRNAs already described as playing important roles in regulating the induction of type I IFNs. Notably, miR155, induced broadly and strongly in response to IFN, has been shown to both suppress TLR signaling and induce IFN signaling through targeting of SOCS1 (42). This regulation may occur in a cell type-specific manner or act within the same cell to induce a shift to suppress TLR-based signaling and enhance IFN signaling, once the secondary signal has been induced. Alternatively, miR155 induction by IFN in a cell where TLR induction has not yet occurred could render a cell unresponsive to TLR signaling and focus cell resources into the induction of an IFN-mediated protective state. Overall, the existing data suggest a negative regulatory role for the major miR155 strand in buffering TLR signaling that may be induced either through NF-κB signaling or downstream of IFN signaling. This contrasts the role for the minor miR155* strand in enhancing IFN signaling through negative regulation of IRAK3, described previously, suggesting an important miR155 dependent switch in immune response.

**Table 1:** **Table of known IFN-regulated microRNAs**.

MicroRNA	Stimulation	Cell type/tissue	Change	Technique	Reference
Let7a	IFNβ	Glioma	Down	Microarray	(52)
Let7b	IFNα	Huh7	Up	Microarray	(55)
	IFNβ	Glioma	Down	Microarray	(52)
Let7c	IFNβ	Glioma	Down	Microarray	(52)
Let7f	IFNα	Huh7	Up	Microarray	(55)
miR1	IFNα	PBMC	Up	RT-PCR	(53)
	IFNβ	Huh7	Up	Microarray, RT-PCR	(50, 56)
		Primary hepatocyte	Up	RT-PCR	(50)
miR100	IFNβ	Glioma	Down	Microarray	(52)
miR122	IFNβ	Huh7	Down	Microarray	(50)
miR1225	IFNα	Huh7	Down	Microarray	(55)
miR128	IFNα	PBMC	Up	RT-PCR	(53)
	IFNβ	Huh7	Up	Microarray, RT-PCR	(50, 56)
miR129	IFNβ	HeLa	Up	Microarray	(57)
				RT-PCR	
miR1296	IFNα	Huh7	Down	Microarray	(55)
miR142	IFNβ	Huh7	Up	RT-PCR	(56)
miR143	IFNα	Huh7	Up	Microarray	(55)
miR146	IFNβ	Huh7	Up	RT-PCR	(56)
miR155	IFNβ	BMM	Up	Microarray	(26)
	IFNβ	RAW264.7	Up	RT-PCR	(58)
	IFNβ	Huh7	Up	RT-PCR, Microarray	(50, 56)
		Primary hepatocyte	Up	RT-PCR	(50)
miR181a	IFNα	Huh7	Up	Microarray	(55)
miR184	IFNα	Huh7	Down	Microarray	(55)
miR187	IFNβ	Glioma	Up	Microarray	(52)
miR190b	IFNα	Huh7	Down	Microarray	(55)
miR194	IFNβ	Glioma	Up	Microarray	(52)
miR195	IFNβ	LX-2	Up	RT-PCR	(59)
miR196a	IFNβ	Huh7	Up	RT-PCR	(56)
miR21	IFNβ	Glioma	Down	Microarray	(52)
miR212	IFNα	Huh7	Down	Microarray	(55)
miR296	IFNβ	Huh7	Down	RT-PCR	(56)
	IFNβ	Huh7	Down	Microarray	(50)
miR30	IFNα	PBMC	Up	RT-PCR	(53)
	IFNα	Blood-derived human NK cells	Down	Sequencing	(54)
	IFNβ	Huh7	Up	Microarray, RT-PCR	(50, 56)
miR301	IFNα	Huh7	Up	Microarray	(55)
miR34a	IFNβ		Up		(60)
miR351	IFNβ	Huh7	Up	Microarray	(50)
miR378	IFNα	Blood-derived human NK cells	Down	Sequencing	(54)
miR431	IFNβ	Huh7	Up	Microarray	(50)
miR448	IFNβ	Huh7	Up	Microarray	(50)
miR449a	IFNα	Huh7	Down	Microarray	(55)
miR499a	IFNα	Huh7	Up	Microarray	(55)
miR518b	IFNα	Huh7	Down	Microarray	(55)
miR582	IFNα	Huh7	Up	Microarray	(55)

*List of reported IFN-regulated microRNAs, identified through specific cellular stimulation with type I IFN*.

IFNβ is currently used as a therapy for multiple sclerosis (MS). In about 85% of patients, disease associated with MS starts with a single demyelinating episode (clinically isolated syndrome, CIS), which progresses to a relapsing-remitting course (RRMS) with acute exacerbations and periods of remission. A study by Hecker et al. longitudinally examined microRNA expression profiles in PBMCs from patients with CIS or RRMS in response to subcutaneous IFNβ therapy (61). Microarray analysis demonstrated seven microRNAs were up-regulated (e.g., let7a, let7b) and 13 microRNAs were down-regulated (e.g., miR29a, miR29c) following IFNβ treatment. Consistent with these results, miR29 has been identified in our unpublished studies as up-regulated early by IFNβ, yet down-regulated by 48 h. Given the known role of miR29 targeting IFNAR1, this regulatory relationship suggests a negative feedback role in limiting the type I IFN response. Such a relationship would provide a capacity for a cell to rapidly induce the IFN response, while providing innate protection against the detrimental impacts of over-activation or an inappropriately sustained response.

## IFN Regulation of MicroRNA Machinery

In addition to direct regulation of microRNAs by IFN, modulation of the microRNA processing machinery would be predicted to have wide-scale impacts on the overall biological outcome. Examination of IFN-mediated transcript regulation through analysis with the Interferome database (a global collection of IRGs) (5) suggests strong down-regulation by more than threefold in both Ago1 and Ago2 in lung and blood cells 24 h following IFNα treatment (62, 63). Emerging evidence suggests that microRNA may have differential association with the various Argonaute family members (64). This relationship introduces a possibility that differential down-regulation of Ago proteins may act as an additional IFN-induced, regulatory mechanism. In the absence of Ago1 and 2, it could be expected that IFN stimulation would favor activity of microRNAs that predominantly interact with Ago3 and Ago4. In addition to the Argonaute protein regulation, evidence exists for post-transcriptional Dicer down-regulation with prolonged IFN stimulation. Using Western blot, Weisen et al. demonstrated that both IFNα and polyI:C stimulation could lead to down-regulation of Dicer after 72 h (65). These longer-term regulatory impacts of the type I IFN response on the microRNA cellular machinery suggest a biphasic response in which early microRNA regulation plays a key role in determining cellular responses and protection. By contrast, longer-term suppression of microRNA regulation may provide a benefit by preventing hijacking of the system by pathogen-derived microRNA. In addition to broad scale changes in regulatory machinery, specific changes in microRNA targeting may be controlled through the adenosine deaminase (ADAR) or apolipoprotein B mRNA editing enzyme, catalytic polypeptide (Apobec) families of proteins, both of which are induced strongly by IFN. ADAR family members, particularly, are widely reported to direct microRNA regulation through transcript editing and warrant further investigations in an IFN context (66).

## Interferon Regulation of Anti-Viral MicroRNAs

The anti-viral functions of effector proteins induced during the IFN response are well understood and studies now focus on the role of IFN-induced microRNAs in direct targeting of viral transcripts (50, 67). It is hypothesized that this could occur in a manner similar to the well-characterized, RNAi-based, plant anti-viral defense system (68). While this hypothesis remains contentious, growing evidence exists for direct viral targeting by cellular microRNAs. This includes investigations in viral infections, including hepatitis B (69), hepatitis C (50, 70), and HIV (71). These studies have identified miR122 as targeting hepatitis B and C and miR29 as targeting HIV; both miR122 and miR29 have been reported previously as IFN-regulated microRNAs. These data suggest that IFN-induced microRNAs may directly target viral RNA in addition to modifying the cellular state through regulation of host anti-viral genes. This targeting strategy is also being investigated for the development of experimental vaccines against influenza A virus. Through the insertion of mammalian-specific microRNA target sites in the viral genome, egg-produced live viruses can be rendered attenuated through microRNA silencing in mammalian vaccine recipients (72). The breadth of IFN-regulated host microRNAs that may directly target viral RNA remains to be determined.

## Identification of MicroRNA Targets

To further understand the role of known IFN-regulated microRNAs and those which regulate the IFN response, predicting the structure and functional significance of regulatory networks, identifying novel microRNAs, and understanding the targeting relationships is critical. Accurate computational prediction of microRNA regulation remains limited and represents an active area of research. In 2005, Brenecke et al. characterized three classes of microRNA binding (73):

1. Canonical binding involved high complementarity throughout the sequence with exact complementarity observed within the last six to eight base pairs of the 5′ end of the microRNA, termed the “seed” region.
2. 5′ dominant seed region binding was defined where the seed region exhibited high complementarity, while the remaining microRNA had limited complementarity with the target region.
3. 3′ compensatory binding was described in which binding in the 3′ region can compensate for mismatches in the 5′ seed region (73).

These definitions were further expanded in 2009, resulting in the definition of seven types of sites: five based on seed region complementarity resulting in seed based binding sites plus two additional categories, 3′ supplementary and 3′ compensatory (74). The seed-based matches are composed of three canonical sites that vary in length from seven to eight base pairs and differ by the association with an adenine at the 5′ end of the microRNA (75). A further two seed-based sites with a six base pair region complementarity have also been identified; however, due to their frequency, these are rarely detected using algorithmic approaches. The 3′ site categories remain similar to those previously defined, with supplementary sites containing consecutive base pair complementarity at the 3′ end. In the 3′ compensatory category, binding in the 3′ region acts to negate mismatches in the seed region. Indeed, many studies have now shown conclusively that binding is more complex than simple seed region recognition. Fluorescence reporter assays have been applied to validate seedless target recognition, while sequence-based, high-throughput target validation has demonstrated the diversity of these interactions (76–79).

As the definitions of these sites have improved, the availability and diversity of algorithms for their detection have also increased. Algorithms for microRNA target site identification include miRanda (80), Dianna-microT (81), PicTar (82), PITA (83), and RNA22 (84). Despite this diversity of algorithms, the ability to predict targets that can be experimentally validated is limited, with a high frequency of false positive results being the common problem. Indeed, comprehensive algorithm comparisons suggest sensitivity rates, defined as the number of correctly predicted sites as a proportion of total correct sites, to vary between 6 and 20% depending on the algorithm applied (85). Equally, precision, defined as the number of correct predictions as a proportion of the total predicted, ranged from 24 to 51% (85). Given the resource investment associated with experimental validation of these interactions, such a poor accuracy rate in computational predictions represents a significant area of concern. These numbers, however, have not been reliably and extensively determined specifically for microRNAs involved in innate immune or IFN responses. As such, for these biological systems, the breadth of IFN-regulated microRNA target networks remains to be determined.

Wide-scale experimental mapping of microRNA binding sites is emerging as the solution to these limitations. These methods include PAR-CLIP (86), HITS-CLIP (87) and CLASH (88), which involve UV or chemical crosslinking, and the use of antibody based methods to pull down the RISC complex and associated microRNAs, and target transcript RNAs. When combined with high-throughput sequencing, the resulting samples can provide a detailed overview of microRNA binding to a target within a cell. While much like early chromatin immunoprecipitation-based transcription factor analysis, antibody efficiency and protocol sensitivities currently limit widespread adoption of these techniques. Nevertheless, these methods provide great potential for future understanding of microRNA networks and their regulation.

## MicroRNA Targeting of IFN-Regulated Genes

While there are limited direct studies of IFN-regulated microRNA targeting of IRGs, one of the first applications of the HITS-CLIP approach compared activated CD4^+^ T-cells in wild-type and miR155 knockout cells from mice, and provided indirect evidence that this microRNA targeted IRGs (79). This analysis identified 4195 genes containing Ago-binding sites, of which 175 genes were predicted to be regulated by miR155. This analysis identified microRNA binding sites previously predicted by computational methods, but approximately 40% of the experimentally identified sites lacked perfect seed complementarity, and thus could not have been predicted computationally. Interestingly, meta-analysis of the 175 genes predicted to be regulated by miR155 included 82 genes that were contained within the Interferome database (5) as IRGs (1.5-fold cutoff). Analysis of this first set of immune-related microRNA targeting relationships supports a role for inducible miR155 targeting, suggesting that miR155 acts as both as an IFN inducible microRNA and a negative regulator of the type I IFN response in the context of CD4^+^ T-cell activation (79). Microarray based correlation analyses have also suggested a relationship between IFNβ inducible miR128, miR196a, or miR142 with reported IRGs HNMT, XPO1, PMPCB, and HMGB1 (56). However, evidence of multiple microRNA directly targeting IRGs remains to be presented. As these RNA-immunoprecipitation based technologies become more readily available, examination of the type I IFN response, specifically, and the innate immune response, more broadly, will elucidate the importance of microRNA targeting as a component of these responses.

## Summary, Conclusion, and Future Directions

Recent studies have shown an important role for microRNAs in regulating the innate and adaptive immune response, and key cytokines in these responses, including the type I IFNs. There are examples of microRNA regulation at many stages of the IFN response, namely through regulation of components of PRR signaling that drive IFN expression; their cognate receptor components, IFNAR1 and IFNAR2; down-stream signal transduction pathways including STATs; and through association with IRGs themselves. There are currently 36 microRNAs reported to be regulated by type I IFNs; but with improvements in sequencing technologies, we can expect this number to grow substantially (as we have seen in unpublished studies). Not only do IFN-regulated microRNAs target components of the IFN response to modulate its biological effects, such as anti-viral actions, they can also directly target viral RNA. In addition to regulating the transcription of microRNAs, IFN may also show unique regulation of microRNA processing by regulation of Dicer, Ago, and editing proteins, which are themselves IRGs. Thus, part of the IFN response may be a general impact on microRNA processing. Advances in technologies such as CLIP, combined with RNA sequencing, will enable the further definition of the breadth of microRNA regulation of IFN responses in different contexts. Given the capacity of microRNA networks to “buffer’ responses, their modulation may open new therapeutic opportunities. Finally, given the use of microRNA detection as biomarkers in cancer, there may be similar opportunities in inflammatory diseases and numerous previously described IFN-mediated conditions, including autoimmune diseases such as SLE and MS.

## Author Contributions

SF, MT, and PH all made substantial contributions to the conception and writing of this review. They all provided different components of the important intellectual content, approve this version of the article for publication, agree to be accountable for all aspects of the work, and will ensure that questions related to the accuracy or integrity of any part of the work are appropriately investigated and resolved.

## Conflict of Interest Statement

The authors declare that the research was conducted in the absence of any commercial or financial relationships that could be construed as a potential conflict of interest.
